# Early-Life Exposures, Neurodevelopment, and Health Outcomes: Protocol for a Birth Cohort Study

**DOI:** 10.2196/78593

**Published:** 2026-02-11

**Authors:** Tor A Strand, Maria Averina, Kjersti Sletten Bakken, Sudha Basnet, Yvonne Böttcher, Sandra Huber, Mari Hysing, Ingrid Kvestad, Torben Lüders, Adrian McCann, Dhiraj Pokhrel, Suman Ranjitkar, Arun K Sharma, Merina Shrestha, Manjeswori Ulak, Ram K Chandyo

**Affiliations:** 1 Innlandet Hospital Trust Lillehammer Norway; 2 Centre for International Health Faculty of Medicine University of Bergen Bergen Norway; 3 Department of Laboratory Medicine University Hospital of North Norway Tromsø Norway; 4 Department of Clinical Medicine The Arctic University of Norway Tromsø Norway; 5 Department of Pediatrics Maharajgunj Medical Campus Institute of Medicine Kathmandu Nepal; 6 Clinical Molecular Biology, EpiGen Institute of Clinical Medicine University of Oslo Oslo Norway; 7 EpiGen Medical Division Akershus University Hospital Lørenskog Norway; 8 Department of Psychosocial Science Faculty of Psychology University of Bergen Bergen Norway; 9 LEADERS Nepal Kathmandu Nepal; 10 Siddhi Memorial Hospital Bhaktapur Nepal; 11 Kathmandu Medical College Kathmandu, Bagmati Province Nepal

**Keywords:** Nepal, Developmental Origins of Health and Disease, DOHaD, early child development, nutrition, environmental pollutants

## Abstract

**Background:**

Negative early-life exposures, particularly during the first 1000 days of life, may disrupt organ development and lead to lifelong negative health consequences.

**Objective:**

Using an exposome and deep phenotyping framework, this study aims to characterize established early-life risk factors, including environmental pollutants and nutritional status during pregnancy and infancy, and identify associated short- and long-term health and developmental outcomes.

**Methods:**

We leverage a pregnancy cohort of 800 mother-infant pairs in Bhaktapur, Nepal, nested within a randomized controlled trial (ClinicalTrials.gov: NCT03071666) that evaluated daily vitamin B12 supplementation from before 15 weeks of gestation until 6 months post partum. The primary outcomes of the original trial were linear growth and neurodevelopment at 12 months. In this follow-up, children will be evaluated up to school age to obtain more robust estimates of long-term health outcomes. Exposures include clinical, dietary, cognitive, demographic, and anthropometric variables during pregnancy and infancy, as well as analyses of environmental pollutants, inflammation, micronutrient status, and hormonal status. Outcomes comprise neurodevelopment, morbidity, mental health, vaccine responses, thyroid function, growth, body composition, lung function, and biomarkers of health and development. Our main research questions for this phase of the project are: (1) what are the most common environmental pollutants among Nepalese women and children? (2) Is there a social gradient in exposure to these pollutants? (3) To what extent are these exposures associated with nutritional status, growth, neurodevelopment, and clinical outcomes? Associations will be examined using cross-sectional, case-control, and cohort designs applying advanced statistical methods to address confounding and complex exposure patterns.

**Results:**

Enrollment began in March 2017, and the first child was born in August of the same year. More than 90% of the original cohort (734/800, 91.8%) have provided data up to the children’s fourth birthday. By December 2025, the project will have funding until July 2027, and the papers addressing the main research questions will be submitted for publication before the end of 2026.

**Conclusions:**

This study draws on a well-characterized mother-child cohort in a South Asian setting with repeated biological samples from blood, breast milk, and urine and extensive high-quality longitudinal data on health, growth, and neurodevelopment. By integrating data on environmental exposures, nutrition, inflammation, and biological responses, the project aims to improve understanding of early-life determinants of health and inform policies and potential interventions to protect vulnerable women and children in marginalized settings. While the exploratory nature of exposome analyses entails a risk of spurious associations, careful interpretation and transparent communication of uncertainty will be prioritized.

**International Registered Report Identifier (IRRID):**

DERR1-10.2196/78593

## Introduction

### The Golden 1000 Days

The period from conception until the second birthday is considered crucial for normal organ development and growth [1]. For example, these first 1000 days of life constitute a period of massive and finely tuned brain development in which the brain is particularly vulnerable to adverse influences [2]. Negative exposures in early life not only may have immediate apparent effects but can also result in developmental cascades or “snowball” effects, where small deficits in brain development can become larger over time and spread to other cognitive domains [3]. Ensuring good health and growth in early life is important to secure optimal functioning throughout the life span [4].

### Environmental Pollutants and Health

We are exposed to environmental contaminants through the air, water, food, and many consumer products such as cosmetics and plastics. Environmental pollutants include persistent and nonpersistent substances, for example, polychlorinated biphenyls, organochlorine, and other pesticides; polybrominated diphenyl ethers; per- and polyfluoroalkyl substances (PFAS); heavy metals; phthalates; and bisphenols [5]. Even at low levels, these exposures have raised substantial concerns because they may adversely affect our health, including growth, development, and immunity [5,6]. For example, a study from Denmark found that prenatal PFAS exposure was associated with an increased risk of children being hospitalized for infectious diseases [7]. Another study from the Faroe Islands found that each doubling of plasma PFAS concentration was associated with up to 40% reduction in the concentrations of antibodies to common childhood vaccines [8]. An association between PFAS exposure and poor vaccine response has also been found in low- and lower-middle–income country children [9], which could explain why many vaccines underperform in such settings.

Altered immunity, poor vaccine responses, and increased susceptibility to infections are particularly critical for marginalized children. Unfortunately, environmental pollutants are likely to have disproportionate effects across socioeconomic strata [10], adding to the already high burden of disease among the poorest children of the world. Exposure to many environmental pollutants has also been linked to poor development [5]. There is, for instance, ample evidence of a relationship between lead or mercury exposure and impaired neurodevelopment [11], and a recent study found a link between poor infant motor development and elevated PFAS concentration [12]. However, the evidence is conflicting, possibly due to poor study quality, such as not capturing confounding and interacting factors or poor specificity of the exposures and outcomes [5]. It is also important to remember that the effects of environmental pollutants on growth or development need not be direct. These substances can act indirectly through impaired immunity, increased inflammation, reduced nutrient availability, or disrupted hormonal pathways such as thyroid hormone production. Repeated infections, inflammation, oxidative stress, and deficiencies of specific nutrients are linked to an increased risk of poor growth and development and represent potential mediators for the effects of toxic exposures [13-15]. The simultaneous manifestation of both undernutrition and overweight is increasing, particularly in poor populations [16]. This increase in the double burden of malnutrition has been attributed to access to cheap, ultraprocessed foods; micronutrient deficiencies; and other nutrition-related factors. However, environmental pollutants are also associated with alterations in childhood growth and could contribute to this double burden of malnutrition [17].

### Environmental Pollutants in Nepal

There are many studies highlighting concerns related to the presence of environmental contaminants in indoor dust [18], groundwater [19], rice [20], and soils [21] in Nepal. However, there are not many studies that have described environmental pollutant levels in humans. A recent review on environmental health in Nepal concluded that it is essential to conduct more studies on the consequences of environmental pollutants as they are of great concern and the knowledge is sparse [22].

### Diet, Nutrition, and Environmental Pollutants

Inadequate nutrition has consequences for child health and development [23]. Micronutrient deficiencies may interact with and aggravate the impacts of environmental pollutants. This can be exemplified by 2 recent studies suggesting that folic acid mitigates the adverse health effects of air pollutants and pesticides in relation to neurodevelopmental disorders [24,25]. Moreover, the risk of thyroid disease from iodine deficiency increases in the presence of endocrine disruptors such as perchlorate, thiocyanate, and nitrate [26]. Crucially, impaired thyroid function in early life has catastrophic consequences for the developing brain. Excess intake of nutrients also poses a health threat. For example, iodine excess results in the same health consequences as iodine deficiency, and high intakes of macronutrients increase the risk of overweight and obesity.

### Other Factors Affecting Child Health and Development

In addition to the aforementioned factors, many poverty-related variables can influence a child’s development. These include but are not limited to a child’s home environment, maternal psychological distress, and responsive stimulation [27]. Many of these factors can act as important confounders of the association between exposures and outcomes and, accordingly, are essential to capture when estimating the effects of environmental pollutant exposure.

As described above, exposure to environmental pollutants is of great concern, especially during early life. As new pollutants continuously enter our environment, describing current exposure levels among pregnant and lactating women and infants and the consequences of such exposures is needed for targeted prevention strategies. Obtaining data from studies or biobanks within health facilities would enable the monitoring of changes and rapid identification of new and emerging concerns. Recent reviews have also called for more research on the relationships between cocktails of environmental pollution, nutritional status, and health in vulnerable populations such as children and pregnant women in an “exposome approach” [28]. Novel epidemiological and data-driven statistical methods could enable simultaneous assessment of the impact of multiple environmental pollutants and nutritional factors on child health and development.

### Aims and Project Objectives

The main objectives are to identify current exposure to environmental pollutants during pregnancy and infancy and investigate the consequences of these for infant health and development. Our three hypotheses are as follows: (1) infants and pregnant women in Nepal are exposed to several environmental pollutants (hypothesis 1); (2) exposures to environmental pollutants are associated with other risk factors for poor health (hypothesis 2); and (3) pregnant women and infants face several adverse exposures that, alone or in combination, may have consequences for their health and development.

Hypothesis 1 encompasses the following research questions (RQs):

- RQ 1a: what are the most common environmental pollutants found in Nepalese women?
- RQ 1b: what are the most common environmental pollutants found in Nepalese infants?
- RQ 1c: are the environmental pollutants found in the mothers also identified in their children?

Hypothesis 2 encompasses the following RQs:

- RQ 2a: is there a social gradient in the exposure to environmental pollutants?
- RQ 2b: to what extent do exposures to environmental contaminants tend to co-occur?
- RQ 2c: to what extent do exposures to environmental contaminants occur with other risk factors?

Hypothesis 3 encompasses the following RQs:

- RQ 3a: is exposure to environmental pollutants related to executive functioning and general abilities?
- RQ 3b: is exposure to environmental pollutants associated with poor vaccine response in children?
- RQ 3c: are environmental pollutants related to thyroid function in mothers and children?
- RQ 3d: is exposure to environmental pollutants associated with stunting and underweight?
- RQ 3e: to what extent is exposure to environmental pollutants associated with maternal and child overweight?
- RQ 3f: are environmental pollutants related to impaired lung function at school age in children?
- RQ 3g: is there a recognizable gradient of lung function in children exposed to increasing concentration of environmental pollutants?

## Methods

### Setting, Participants, and Enrollment Procedures

#### The Exposome Concept

The exposome concept includes the integration of (1) the measurement of several exposures in the human external environment, (2) the measurement of a broad spectrum of biological responses in the human body’s internal environment, and (3) addressing the exposome’s dynamic life cycle nature [29]. The exposome approach may lead to a greater understanding of the role of environmental risk factors in child health, growth, and development. Indeed, a recently completed population-based exposome study using data from 6 European countries highlighted the need for more well-characterized cohorts to measure the association between toxic exposures and health outcomes [30].

Because of the high plasticity and vulnerability of the brain during the first 1000 days of life, measurements of development during this period are less reliable than those later in life. Furthermore, for specific domains and functions, such as scores on general abilities (IQ), the predictive ability for future academic achievements increases with age, and scores should preferably be obtained after 4 to 5 years. Taken together, a longitudinal design that investigates exposures during the critical first 1000 days and neurodevelopmental outcomes during preschool is warranted to capture seemingly small but important consequences for the brain. It is also important to measure several potentially mediating factors such as inflammation and infections to not overlook possible mediators or important consequences of environmental exposures.

The RQs will be addressed in a pregnancy cohort with 800 mother-infant pairs in Bhaktapur, Nepal. This cohort is nested within a randomized controlled trial on daily vitamin B12 supplementation during pregnancy. The latter study was designed to address other objectives in a cohort design until the age of 2 years in the children. Details on data collection, funding, and primary objectives have been published elsewhere [31,32].

**Figure 1:**
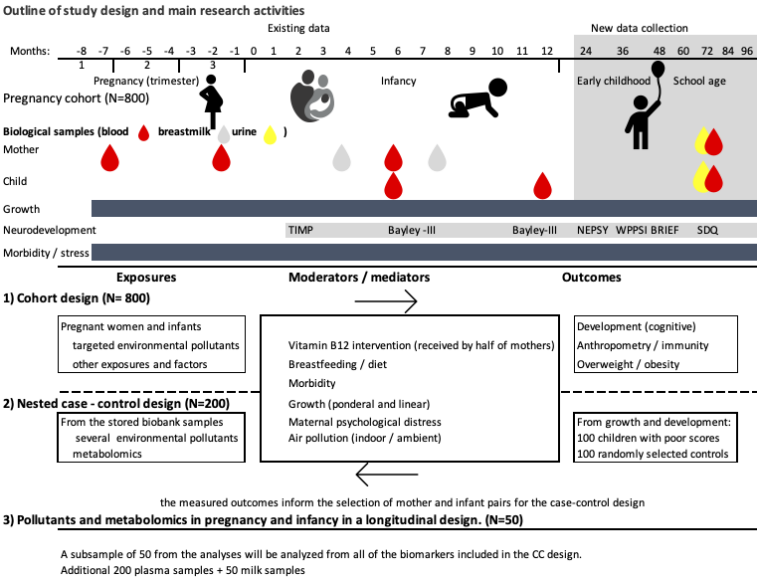
Outline of the study design in a cohort study in Bhaktapur, Nepal. Bayley-III: Bayley Scales of Infant and Toddler Development, third edition; CC: case-control; SDQ: Strengths and Difficulties Questionnaire; TIMP: Test of Infant Motor Performance; WPPSI: Wechsler Preschool and Primary Scale of Intelligence, fourth edition.

The RQs for hypotheses 1 and 2 will be addressed using cross-sectional designs, whereas for the RQs for hypothesis 3, we will use cohort and case-control designs (Figure 1). In the cohort design, we will use the entire sample and fewer but targeted biomarkers (see below). In the case-control design, we will additionally analyze the metabolome and include a broader panel of environmental pollutants in 100 high-risk infants and 100 randomly selected controls. The definition of high-risk infants will be determined using neurodevelopmental test scores and growth indexes. A random process will be used to select the controls. In longitudinal analyses, we will undertake the same biomarker assessments repeatedly in a subset of 50 randomly selected mother-infant pairs from the case-control sample (Figure 1). This analysis aims to assess the extent to which biomarkers vary between mother-infant pairs and whether their concentrations differ across pregnancy, infancy, and the postpartum period. At 7 years of age, we will collect new blood and urine samples from the children and their mothers.

#### Biospecimen Collection

The study is being conducted in the Bhaktapur district in the Kathmandu Valley, Nepal. Bhaktapur is a periurban agriculturally based community inhabited by people from all over Nepal and the most densely populated district in Nepal due to recent migration. At the field site, we have access to a fully functional field office staffed with relevant health personnel and field workers. The blood and urine samples, which are collected for analysis of environmental pollutants, are processed immediately and stored at −70 °C locally, during transport, and in biobanks in Norway.

The study population has already been well described, including variables related to socioeconomic status; mental health; maternal cognitive abilities; vaccination status; air pollution; intrauterine growth; birth outcomes; child, maternal, and paternal anthropometry; and others.

#### Mothers and Infants

Venous blood samples were collected from the mothers at enrollment, 8 months of gestation, and 6 months after delivery and from the infants at 6 and 12 months. Approximately 3 to 4 mL of blood were collected into vials containing ethylenediaminetetraacetic acid. The hemoglobin concentration was estimated immediately using the HemoCue Hb 201 system calibrated per the manufacturer’s guidelines. Blood was centrifuged at approximately 2000 g at room temperature for 10 minutes, and plasma was stored at −70 °C at the field site and always transported on dry ice. We also collected blood samples from the mother-child pairs when the children were aged 7 years. These samples were analyzed for glycated hemoglobin and hemoglobin immediately following collection and stored at −60 or −80 °C in our biobank in Nepal.

### Analyses of Environmental Exposures

Fully validated analytical methods for analyzing environmental pollutants, heavy metals, and trace elements will be applied in the Environmental Pollutant Laboratory at the University Hospital of North Norway. This laboratory uses automated sample preparation methods that can handle small sample volumes for a broad range of analyses. The instrumental analysis will be performed using gas, ultrahigh-performance liquid, and supercritical fluid chromatography combined with different ionization techniques (ie, atmospheric pressure, electrospray, and UniSpray ionization) and coupled to tandem mass spectrometers. The analytical methods for persistent organic pollutants and PFAS in plasma are well established [33]. A multielement method (>30 elements) based on dilution and direct introduction to the inductively coupled plasma mass spectrometer for plasma, whole blood, and urine will be used at the University Hospital of North Norway. This analysis will yield both toxic elements (such as heavy metals) and micronutrients.

### Targeted Metabolomics

We will choose targeted over untargeted metabolomics because untargeted or discovery-based metabolomics focuses on global detection and relative quantitation of metabolites in a sample and, as a result, typically captures only abundant metabolites. The targeted metabolic profiling will include both direct and functional markers of nutritional status (including B vitamins such as B6 and folate; total homocysteine; and the fat-soluble vitamins A, D, E, and K), as well as other nutrients, including essential amino acids. Central to investigating the effect of exposure to environmental pollutants on growth, development, and health outcomes, we will also target a wide array of complementary biomarkers and proxy markers linked to inflammation (eg, C-reactive protein, serum amyloid A, calprotectin, neopterin, the kynurenine-to-tryptophan ratio, and the PAr index of vitamin B6 catabolism) [34-36]. In addition, we will target metabolites involved in defined metabolic pathways related to nucleotide synthesis, methylation reactions, mitochondrial function, energy homeostasis, and amino acid metabolism, including one carbon metabolism, the tricarboxylic acid cycle, and the kynurenine pathway. The targeted metabolomic analyses will be conducted using a methodology based on gas chromatography–tandem mass spectrometry, liquid chromatography–tandem mass spectrometry, and matrix-assisted laser desorption–ionization time-of-flight mass spectrometry (proteins and their proteoforms) and microbiological assays at Bevital AS.

### Gut Microbiome–Derived Metabolites

Gut-derived metabolites in maternal and infant blood will also be measured. These include tryptophan metabolites, the indoles, and short-chain fatty acids, as well as choline derivatives that are produced by gut bacteria. These microbiome-derived metabolites play a key role in immune function, brain development, and metabolic health [37-39] and are invaluable for understanding how maternal and infant gut microbiomes shape long-term health and disease trajectories. Again, targeted metabolomic analyses will be used to reliably detect and quantitate systemic concentrations of these low-abundance metabolites using gas chromatography–tandem mass spectrometry and liquid chromatography–tandem mass spectrometry methods at Bevital AS.

### Telomere Length

Genomic DNA were isolated from whole-blood samples from both mothers and infants (all time points) using an in-house–developed method for automated isolation on the QIAsymphony system (Qiagen). Telomere length was measured using real-time polymerase chain reaction [40]. The 36B4 was used as a reference gene as described previously [41]. Analyses were carried out on a QuantStudio 6 Pro real-time polymerase chain reaction system (Applied Biosystems). Telomere length analyses were performed at the Department of Clinical Molecular Biology (EpiGen), a joint research facility between Akershus University Hospital and the University of Oslo.

### Neurodevelopment: Early Child Development, Cognitive Development, and Mental Health

Figure 1 provides a brief account of neurodevelopmental assessments already conducted in the cohort and assessments up to the age of 7 years. Neurodevelopmental outcomes were assessed using state-of-the art and age-appropriate instruments. At approximately 3 months, the Test of Infant Motor Performance was used to measure infant motor development. The Bayley Scales of Infant and Toddler Development, third edition (BSID-III), were used to assess early child development at 6, 12, 24, and 36 months. The BSID-III have previously been shown to have good psychometric properties at our field site and are widely used worldwide to assess development in young children [42].

For the follow-up, we have also included assessment of specific cognitive functions such as executive functioning through the NEPSY neuropsychological test battery [43] and the parent-reported Behavior Rating Inventory of Executive function–Preschool Version at 4 and 5 years [44]. We also included the Wechsler Preschool and Primary Scale of Intelligence, fourth edition, to measure intellectual functioning in the children at 4 and 5 years. As a measure of cognitive function, we also included a digital tool when the children are aged 6 years. The Developmental Assessment on an E-Platform is a cognitive ability assessment tool for children covering 6 cognitive domains presented to children on a tablet with a gamified approach.

Mental health outcomes are assessed using the BSID-III Social-Emotional scale via interview with caregivers when the children are aged 6 to 36 months. In the follow-ups at 4, 5, and 7 years, the parent-reported Strengths and Difficulties Questionnaire, a mental health screening questionnaire for children, has been included, and at 7 years, the Autism Spectrum Quotient [45], a screening tool for autism spectrum disorders, is included. The latter screening tool is included as autism spectrum disorders have previously been found to be associated with neurotoxic exposures [34]. In this study, we have also included a child health sleep questionnaire to measure the quality and quantity of child sleep at 4 and 7 years.

### Maternal Mental Health, Sleep, and Functioning

From the cohort, there are repeated measures of maternal psychological distress using the Self-Reporting Questionnaire–20 and the Reduced Tension Checklist and of maternal sleep through the Bergen Insomnia Scale. We have included the Raven Progressive Matrices as a measure of maternal nonverbal intellectual functioning. As measures of the quality of parental caregiving, we have included the Home Observation for Measurement of the Environment inventory and the Caregiver Knowledge of Child Development Inventory. These measures will be included in the statistical analyses as potential moderators or mediators. A comprehensive list of the variables included in the project is provided in our study web page [46].

### Vaccine Response

Vaccine coverage in the selected population is excellent, and our previous studies have shown that nearly all children have received their scheduled vaccine doses. In the parent project, all children also received an oral rotavirus vaccine at 6 and 10 weeks. We will measure plasma anti–rotavirus immunoglobulin A antibodies using an enzyme-linked immunosorbent assay in samples drawn at 6 and 12 months. We will also include other vaccine responses (such as measles and polio) as outcomes using enzyme-linked immunosorbent assay methods. For these analyses, we will use the clinical laboratories of the partner institutions (see below) in Nepal.

### Thyroid Function

The markers of maternal thyroid function during early pregnancy have already been measured at the Research Laboratory of the Department of Clinical Biochemistry, Institute of Medicine, Tribhuvan University, Kathmandu, Nepal. Plasma concentrations of free triiodothyronine, free tetraiodothyronine, total triiodothyronine, total tetraiodothyronine, thyroid-stimulating hormone, thyroglobulin, thyroglobulin antibody, thyroid peroxidase antibody, thyroid-stimulating hormone receptor antibodies, and total human chorionic gonadotrophin were analyzed using a Maglumi 800 chemiluminescence auto-analyzer (Snibe Diagnostic).

### Anthropometry (Growth and Body Composition)

Dedicated staff have measured length, height, weight, middle upper arm circumference, and head circumference in the children monthly for the first year of life and biannually thereafter. We will obtain anthropometric measurements biannually at least until 84 months after birth.

### Lung Function

Lung function will be assessed in the original child cohort using spirometry and oscillometry. All children are of school age and can perform reliable spirometry, which will be conducted using open-circuit spirometers in a child-friendly setting with infection control measures. Incentive spirometers will encourage best efforts, and calibration will be done daily with a 3-L syringe.

Testing will be delayed if children recently took bronchodilators (6 hours), had a large meal (2 hours), or exercised (30 minutes). Children will wear light, nonrestrictive clothing; sit comfortably on a stool; and use a nose clip.

Each child will undergo pre- and postbronchodilator oscillometry followed by spirometry. Oscillometry will involve 5 sequences of tidal breathing (≥30 seconds). Following oscillometry, the child will complete at least 3 acceptable, repeatable forced expiratory maneuvers for spirometry, with rest breaks as needed. Up to 10 attempts may be made unless the child tires. After the prebronchodilator test, 400 μg of salbutamol will be administered via metered-dose inhaler using a spacer. The sequence will be repeated 15 to 20 minutes later for postbronchodilator measures.

Spirometry will measure forced vital capacity (FVC), forced expiratory volume in 1 second (FEV1), ratio of FEV1 to FVC (percentage), and forced expiratory flow between 25% and 75% of FVC. A minimum of 3 acceptable, reproducible efforts are required. Spirograms must be artifact free, show a good start and end, and meet repeatability criteria (eg, FVC and FEV1 within 150 mL). Measurements will be made under body temperature, pressure, and saturated conditions and interpreted using Global Lung Function Initiative references following American Thoracic Society and European Respiratory Society guidelines.

Impulse oscillometry will use sound waves over tidal breathing with the child seated, the head neutral, the nose clipped, and the cheeks supported. Children will perform 5 sequences of 30-second tidal breathing. Total respiratory impedance, resonant frequency, respiratory system resistance, respiratory system reactance, and reactance area will be measured and interpreted based on published references for oscillometry in children [47,48].

### Data Management

Data management encompasses capture, storage, sharing, and later use of data. Three individuals in Nepal work solely with data integrity during data collection for this project.

### Data Capture

Data were captured electronically using tablets through the iFormBuilder system (Zerion Software [49]) and case report forms (paper forms). All paper forms are entered twice into a Microsoft Access database by 2 computer operators, and their entries are compared concurrently for errors.

### Data Storage

Data are stored on secure servers at the field site in Nepal and in Norway. Information that could identify participants is stored on separate servers to ensure their privacy. Data used for statistical analyses are always deidentified. Person-identifiable data are stored on a separate server in Nepal with limited and restricted access. Only the data managers and principal investigators have access to this server.

### Statistical Approach and Sample Size

Within the project group, we maintain a registry of identified RQs that can be addressed within the project. Before an RQ is addressed, we have defined the following steps that should be followed:

1. Define and spell out the RQ.
2. Define a core writing group (CWR) and a list of potential authors and suggest the key positions of the list of authors (first, second, last, and corresponding).
3. The CWR will prepare the first version of the plan of analysis (PoA), which will be circulated and agreed upon among all authors. This plan will define the main exposure and outcome variables and their expected distributions (eg, continuous and categorical). It will also include a description of other variables to be included in the statistical models or of the process for deciding which variables to include. The PoA will also include information on how missing data will be handled and whether and how sensitivity analyses will be undertaken.
4. When the PoA is finalized, it will be uploaded to a suitable repository, such as Zenodo [50] or a clinical trial registry.
5. The CWR will undertake the analyses and present the results and a summary (abstract) of the work for the entire group.
6. The CWR will lead the writing of the final report.
7. Once all suggested authors have had the opportunity to contribute, we will define the final authorship list.

During this process, we will follow established guidelines, such as the Strengthening the Reporting of Observational studies in Epidemiology guidelines and the Vancouver guidelines, when defining who to include in different scientific manuscripts.

To estimate the association between the selected exposures and outcomes, we will use different regression methods. We will explore dose-response relationships using generalized additive models, cubic splines, and similar techniques and measure potential mediation using structural equation models. In addition to the more traditional regression-based approaches, we will evaluate and develop machine learning approaches to address our RQs. The major advantage of machine learning is the ability to deal with the plethora of exposures and the extensive number of combinations; a feature that traditional regression-based methods struggle with. However, the resulting machine learning models can be more challenging to interpret. To develop models that are both interpretable and high performing, we will consider intrinsically interpretable models such as least absolute shrinkage and selection operator regression and provide post hoc explanations for more complex methods such as neural networks.

For most of the RQs, we will have a sample size of 800 participants. This way, we will be able to describe relevant prevalences and exposure-outcome associations precisely. For each RQ, we will calculate the expected precision of the effect measure estimates when preparing the analysis plans. Limited statistical power and precision will be adequately discussed when relevant.

### Ethical Considerations

All phases, including the follow-up described in this manuscript and the measurement of leucocyte telomere length, environmental pollutants, and metabolomics, have been approved by the Nepal Health Research Council (253/2016) and the Regional Committee for Medical and Health Research Ethics in Norway (project 2016/1620/REK vest). After thorough information was provided to the parents, we obtained written informed consent or a thumbprint from illiterate individuals (in the presence of an impartial witness). We keep a registry of all women and witnesses if this is required. Personal identifiable information (such as names, addresses, and dates of birth) will be stored separately from research data on a secure server not connected to the internet and in a locked cabinet with restricted access. Participants may withdraw from the study at any time and request that their data be deleted. The study team will ensure that such requests are complied with in accordance with applicable regulations. Personal identifiable data will not be shared with third parties beyond authorized data handlers involved in the study.

In the consent form, we included a question regarding the use of data for purposes other than the original outcomes of the randomized controlled trial and the use of the biobank for other analyses. Only those who opted in for this purpose will be included in subsequent analyses. When the objectives of the secondary analyses are not covered in the original consent form, we request an updated consent form that includes the new objectives. As of December 2025, most of the participants have completed 4 consent forms. We keep a record of what all participants have consented to since the project started in 2017.

The study participants in this phase of the project were compensated for their travel time to the study site. As part of the main study, participants received a small gift once a year. The maximum value of these gifts is US $50.

## Results

**Figure 2:**
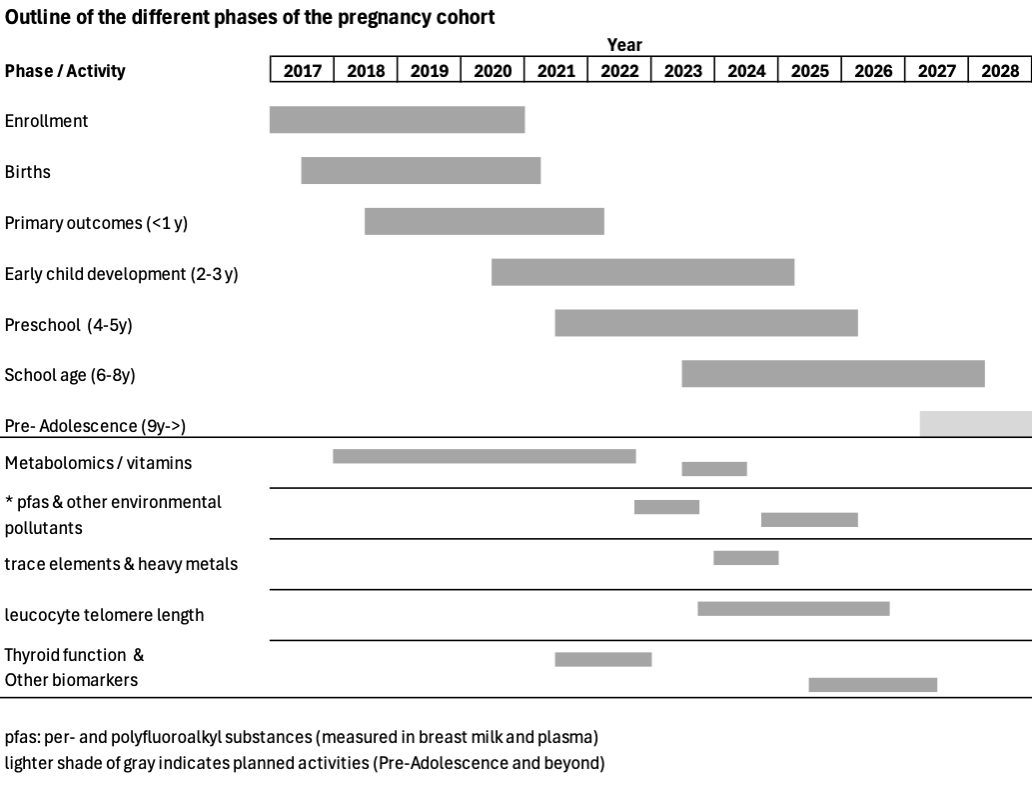
Outline of the different phases of a pregnancy cohort study in Nepal. The lighter shade of gray indicates planned activities (preadolescence and beyond). PFAS: per- and polyfluoroalkyl substances.

As of December 2025, we were still following up with 91.8% (734/800) of the original mother-infant pairs included in the study, and the study is funded until July 2027. We have completed 662, 430, 190, and 20 for the 5-, 6-, 7-, and 8-year follow-ups, respectively. We have also analyzed metabolomic responses, trace element and heavy metal concentrations, and PFAS concentrations from baseline plasma samples in all enrolled women. By May 2026, all children in the cohort will have reached their fifth birthday, and we will have complete follow-up data on key outcomes and exposures for >90% of these participants. The timing of the different phases of the study is shown in Figure 2.

## Discussion

This well-characterized mother-child cohort with extensive longitudinal data and repeated biological samples will serve as a valuable resource for studying exposure to environmental pollutants and nutritional status in a densely populated district in Nepal that includes both rural and urban residents. We will also assess the relationships between these exposures and various health-related outcomes. For instance, data from this project have been used to describe B vitamin and selenium status and examine the relationship between selenium levels and neurodevelopment [51,52].

While the broad scope and detailed phenotyping approach offer valuable opportunities to identify potential determinants of long-term outcomes, many analyses are inherently exploratory and involve multiple correlated exposures and outcomes. As a result, the observed associations should be viewed as hypothesis generating rather than causal. We will focus on effect sizes and uncertainty intervals instead of significance testing with dichotomous *P* values. As *P* values depend on effect size, variability, measurement error, and sample size, they have limited usefulness for communicating uncertainty. Additionally, our findings from this cohort may not directly apply to other populations or settings with different exposure profiles, nutritional patterns, or health systems.

Even when following well-defined and prespecified analysis plans, the large number of associations that can be tested may lead to false positives and spurious results. At the same time, even sample sizes of up to 800 might be too small to detect clinically meaningful associations with the necessary precision. Additionally, variables differ greatly in how effectively they measure what they are intended to capture. For example, we are confident in our anthropometric measurements, including the methods for obtaining them and what they represent; however, many factors can influence our neurodevelopmental assessments, such as the children’s fatigue level, ongoing illnesses, mood, temperament, and current home environment. These factors increase the uncertainty about the interpretation of the findings, which we will carefully discuss when we communicate our results.

The dissemination of results will be conducted carefully and supported by a dedicated communication work package, with designated personnel responsible for engaging media and lay audiences. This communication strategy ensures that both the identified associations and their inherent uncertainties, as well as the contextual nature of the findings, are conveyed accurately, transparently, and without overstating their significance.

## Appendix

Multimedia Appendix 1 Peer review comments from the Research Council of Norway (Forskningsrådet).

## Abbreviations

BSID-III: Bayley Scales of Infant and Toddler Development, third edition
CWR: core writing group
FEV1: forced expiratory volume in 1 second
FVC: forced vital capacity
PFAS: per- and polyfluoroalkyl substances
PoA: plan of analysis
RQ: research question

## Data Availability

The datasets generated or analyzed during this study are available from the corresponding author or the emails below on reasonable request. To meet ethical requirements for the use of confidential patient data, requests must be approved by the Nepal Health Research Council and the Norwegian Regional Ethics Committee. Requests for data can also be sent to the Child Health Research Project, Department of Child Health, Institute of Medicine, Tribhuvan University (chrp2015@gmail.com), or the Department of Global Public Health and Primary Care at the University of Bergen (post@igs.uib.no). Upon completion of the study, we will anonymize the data and share the code and metadata on appropriate servers for further use in accordance with national rules in Norway and Nepal.
