# Supplementary material for: Early-Life Exposures, Neurodevelopment, and Health Outcomes: Protocol for a Birth Cohort Study
Source: JMIR Res Protoc. 2026 Feb 11;15:e78593. doi: 10.2196/78593 (PMC12936664; doi:10.2196/78593)
Supplement: Multimedia Appendix 1 [file resprot_v15i1e78593_app1.pdf]

The Research Council of Norway

Institutt for global helse og samfunnsmedisin
UNIVERSITETET I BERGEN
Postboks 7804
5020 BERGEN
Norge

**Enquiries to**	**Our ref.:**	**Oslo,**
Karine Kålsås	336566/KKA	10.10.2022
+4722037402	**Application received:**	
kka@forskningsradet.no	09.02.2022	

Call for proposals:	Collaborative Project in Global Health, 09.02.2022
Project:	Toxic exposures in early life, growth, and development: An exposome study in Nepal.
Project No.:	336566
Project Owner:	Institutt for global helse og samfunnsmedisin, UNIVERSITETET I BERGEN
Project Manager:	Tor Arne Strand
Project Administrator:	Ingvild Strømsnes Maden

Dear Sir/Madam,

We are pleased to inform you that the Research Council of Norway, by the Portfolio board for global development and international relations, has made a conditional decision to grant up to 12 000 000 for your project, contingent upon the revision of the grant application as stipulated below. Before you revise the grant application, the project is obligated to have a contract meeting with the representative case officer for this project to clarify closer important aspects to be revised before a final project acceptance.

The Research Council is in a demanding financial situation. The Research Council's Executive Board has discussed several different measures to remedy the situation both in short and long term and has asked the administration to ensure that the projects start up later than originally planned. For you, this means that the Research Council cannot finance project costs that accrue in 2022. Furthermore, the project may start when suitable in 2023, but with reduced budget and progress the first year. This means that the total project budget for 2023

Norges forskningsråd/
The Research Council of Norway
Drammensveien 288
Postboks 564
NO–1327 Lysaker

Telefon +47 22 03 70 00
Telefaks +47 22 03 70 01
post@forskningsradet.no
www.forskningsradet.no
Org.nr. 970141669

All post og e-post som inngår i saksbehandlingen, bes adressert til Norges forskningsråd og ikke til enkeltpersoner.

Kindly address all mail and e-mail to the Research Council of Norway, not to individual staff.

may not exceed 50% of the originally planned budget for 2023. We will get back to you with further information in connection with the contract meeting.

A list of the projects awarded funding along with a list of the referees used to assess the grant applications under this call for proposals may be found on the Research Council's website. Please contact the relevant case officer for more information about which experts reviewed your grant application.

Revising the grant application

You need to update or confirm the objectives, progress plan, and items relating to the budget, project description and other relevant components of the grant application.

To revise the grant application, go to “Applications in eSøknad” on My RCN web. The application is now available as “Revised”. You will need to provide additional budget information in the revised grant application, so the application form may look somewhat different from the original application you submitted.

The content of the revised grant application will be used as the basis for the contract between the Research Council and the Project Owner. Read more about the contracts for R&D projects here.

Stipulations:

- Only in special occasions can the project partners be changed. If changes are needed, this must be approved by the Research Council and communicated to existing and new partners before entering into the contract.

- Costs that accrue before 1 January 2023 will not be financed by Research Council funding.

- Please mention, if possible, the name and country of your partner institution(s) in the popular science summary.

- We ask you to read the panel's assessment of the project and consider if comments can give useful improvements to the project.

- We ask you to submit a cover letter explaining any changes to the project or project description made as a result of the expert assessments. If you make changes in the project description, please use the "Track changes" function when editing text. If you find any recommendations from the panel not to be relevant for the project, please provide an explanation of this in the cover letter.

- Send in a completed detailed budget attachment with your revised application. A detailed budget template has been sent to the project manager via email. Please look closely at the guidelines given in the budget template.

- You also need to submit an attachment outlining how the project will ensure that the principles of Equitable partnerships are fulfilled in the project. Please find a template for this in the email sent to the project manager.

- Please make sure that the progress plan is sufficiently specific to make it suitable as a reference to actual project progress. We ask that you check that the progress plan in the application form corresponds with the activities/WPs in the project description. The progress plan should include both main activities and milestones.

- According to the specifications in the cost plan the project plan to purchase ultra-freezers. Please see the guidelines in the online form for more information on how to enter the costs for equipment. You can also find useful information about this here. Please also make sure to specify the costs related to equipment in the detailed budget.

- The working time of Sandra Huber, Maria Averina and Jan Brox will be own contribution. But these expenses have not been included in the total budget. Please include such contributions in the budget if possible.

- All doctoral, post-doctoral candidates and visiting researchers funded by the Research Council must be registered under "Fellowships" in the online project application. Add name (if unknown use NN), start and end dates, man months and country where the work will be conducted.

Provide a popular science presentation

You must write a popular science presentation in both Norwegian and English in your revised grant application. It is important to write both the project summary and the popular science presentation in a way that is understandable to individuals who do not have the relevant scientific background. Make sure you do not include any confidential information from the project in these texts. Please note that information about the project will be published in the Research Council's project databank once the revised grant application has been approved. Read more about the Project Databank and how to write good popular science presentations here.

Required submission of data management plans

In connection with the revision of the grant application, R&D-performing organisations or companies must assess the need to develop a data management plan for all projects that have been granted research funding. These plans are normally required if the project collects or in some other way produces research data. If the Project Owner decides that the project does not need to develop a data management plan, an explanation of this must be provided in the grant application. Information about the archive solution(s) to be used for storing the data is to be

Norges forskningsråd/
The Research Council of Norway
Drammensveien 288
Postboks 564
NO–1327 Lysaker

Telefon +47 22 03 70 00
Telefaks +47 22 03 70 01
post@forskningsradet.no
www.forskningsradet.no
Org.nr. 970141669

All post og e-post som inngår i saksbehandlingen, bes adressert til Norges forskningsråd og ikke til enkeltpersoner.

Kindly address all mail and e-mail to the Research Council of Norway, not to individual staff.

provided in the data management plan. Click here for more information about data management plans.

Collaboration agreements
The Project Owner is responsible for ensuring that written agreements are signed with all the relevant partners taking part in the project. Suggestions regarding elements to include in an agreement may be found here.

A copy of all signed collaboration agreements is to be attached to the contract when this is returned to the Research Council. You must return the contract documents within three months after we have made it available for you on My RCN Web. The contract will not enter into force until the collaboration agreements are in place. Read more about the "General Terms and Conditions for R&D Projects" here.

Information about scientific assessment and allocation decisions
We have enclosed an overview of the review of your proposal with the general mark for your grant application and the marks assigned to each individual criterion. We have also attached the assessments of the panel of referees. These assessments are being forwarded to provide you with scientific feedback, and do not comprise the grounds for the funding decision. Please notice that applicants are not permitted to contact the referees.

Limited right to lodge a complaint
The decision of the Research Council is exempt from provisions of the Norwegian Public Administration Act regarding complaints to a superior agency. However, the Research Council does allow complaints within a restricted framework. For more information, see the Research Council's website. Please note that it is *not* permitted to submit complaints relating to the academic or expert assessments and priorities that form the basis for the decision to reject the application.

The deadline for submitting a complaint is 31. October 2022. Complaints must be submitted in writing and may only be put forth by the Project Owner (institution) via the designated project administrator.

Insider information

If you become aware that information provided to the Research Council comprises insider information in accordance with the Securities Trading Act, we ask you please to inform us of this.

Do you have questions?
Please feel free to contact the case officer Karine Kålsås by email kka@forskningsradet.no if you have any questions. Please be sure to include the reference number 336566 on all enquiries to the Research Council relating to this project.

Yours sincerely,
The Research Council of Norway

Marianne Jensen
Department Director

Karine Kålsås
Case Officer

Approved and expedited electronically without signature

Attachments

Norges forskningsråd/
The Research Council of Norway
Drammensveien 288
Postboks 564
NO–1327 Lysaker

Telefon +47 22 03 70 00
Telefaks +47 22 03 70 01
post@forskningsradet.no
www.forskningsradet.no
Org.nr. 970141669

All post og e-post som inngår i saksbehandlingen, bes adressert til Norges forskningsråd og ikke til enkeltpersoner.

Kindly address all mail and e-mail to the Research Council of Norway, not to individual staff.
